# State space and movement specification in open population spatial capture–recapture models

**DOI:** 10.1002/ece3.4509

**Published:** 2018-09-27

**Authors:** Beth Gardner, Rahel Sollmann, N. Samba Kumar, Devcharan Jathanna, K. Ullas Karanth

**Affiliations:** ^1^ School of Environmental and Forest Sciences University of Washington Seattle Washington; ^2^ Department of Wildlife, Fish, and Conservation Biology University of California, Davis Davis California; ^3^ Centre for Wildlife Studies Bangalore Karnataka India; ^4^ Wildlife Conservation Society – Global Conservation Program Bronx New York; ^5^ National Centre for Biological Sciences‐TIFR Bangalore India

**Keywords:** camera‐trapping, dispersal, Markovian movement, population dynamics, tigers, transience

## Abstract

With continued global changes, such as climate change, biodiversity loss, and habitat fragmentation, the need for assessment of long‐term population dynamics and population monitoring of threatened species is growing. One powerful way to estimate population size and dynamics is through capture–recapture methods. Spatial capture (SCR) models for open populations make efficient use of capture–recapture data, while being robust to design changes. Relatively few studies have implemented open SCR models, and to date, very few have explored potential issues in defining these models. We develop a series of simulation studies to examine the effects of the state‐space definition and between‐primary‐period movement models on demographic parameter estimation. We demonstrate the implications on a 10‐year camera‐trap study of tigers in India. The results of our simulation study show that movement biases survival estimates in open SCR models when little is known about between‐primary‐period movements of animals. The size of the state‐space delineation can also bias the estimates of survival in certain cases.We found that both the state‐space definition and the between‐primary‐period movement specification affected survival estimates in the analysis of the tiger dataset (posterior mean estimates of survival ranged from 0.71 to 0.89). In general, we suggest that open SCR models can provide an efficient and flexible framework for long‐term monitoring of populations; however, in many cases, realistic modeling of between‐primary‐period movements is crucial for unbiased estimates of survival and density.

## 1 INTRODUCTION

Knowledge of animal population size and dynamics is essential to assess a species’ status, inform conservation strategies, and advance ecological understanding. Capture–recapture methods (Otis, Burnham, White, & Anderson, 1978; Pollock, Nichols, Brownie, & Hines, 1990) have traditionally been widely used to estimate these population parameters based on repeated observations of individually identifiable animals. These models produce unbiased estimates of population size and vital rates by correcting for imperfect detection. Capture–recapture models can broadly be divided into two classes. Models for closed populations use multiple sampling events over a short time frame to estimate the size of a static population (Otis et al., 1978). Models for open populations estimate vital rates such as survival and recruitment between sampling periods when the population under study is allowed to change (Cormack, 1964; Jolly, 1965; Pollock et al., 1990; Seber, 1965). Developments have been made to these models to improve parameter estimates including the development of the robust design (Kendall, Nichols, & Hines, 1997; Pollock, 1982), which provides a flexible framework for combining open and closed models.

Another major advancement in capture–recapture modeling was the development of closed spatial capture–recapture models (SCR; Efford, 2004; Borchers & Efford, 2008; Royle, Chandler, Sollmann, & Gardner, 2014). Since their development about 10 years ago, SCR models have become increasingly popular and are widely used in conjunction with a number of survey methods such as camera‐trapping (Efford, Dawson, Jhala, & Qureshi, 2015; O'Connell, Nichols, & Karanth, 2011; Royle, Karanth, Gopalaswamy, & Kumar, 2009), noninvasive DNA sampling (Gardner, Royle, & Wegan, 2009; Whittington & Sawaya, 2015), acoustic detectors (Efford, Dawson, & Borchers, 2009), or live trapping (Gerber & Parmenter, 2015). Contrary to traditional capture–recapture models, which condense detections of individuals across multiple sampling devices into a binary “detected anywhere on the sampling grid” format, SCR models make use of the spatial information of individual detections. In a Bayesian implementation, this information is used to estimate the location of individual activity centers and to describe the probability of detecting an individual at a given trap as a decreasing function of the distance of that trap to the animal's activity center. By employing a model of animal movement and detection, SCR models account for variation in individual exposure to sampling, a source of heterogeneity in detection probability traditional models cannot directly account for. A number of extensions have been developed for closed SCR models including mark–resight models (Sollmann, Gardner, Parsons et al., 2013; Sollmann, Gardner, Shindle, et al., 2013; Whittington, Hebblewhite, & Chandler, 2018), resource selection functions (Royle, Chandler, Sun, & Fuller, 2013), noncircular home ranges (Sutherland, Fuller, & Royle, 2015), continuous time encounter probabilities (Borchers, Distiller, Foster, Harmsen, & Milazzo, 2014; Dorazio and Karanth 2017, spatial variation in density (Borchers & Efford, 2008; Reich & Gardner, 2014; Royle et al., 2014), etc.

Despite development around the same time as closed SCR models, applications of SCR models for open populations (Gardner, Reppucci, Lucherini, & Royle, 2010) have been limited to only a handful of published studies (Chandler & Clark, 2014; Ergon & Gardner, 2014; Raabe, Gardner, & Hightower, 2014; Schaub & Royle, 2014; Whittington & Sawaya, 2015). Estimates of survival from traditional capture–recapture models usually refer to “apparent survival” (the product of survival and the probability of remaining in the sampled area, Lebreton, Burnham, Clobert, & Anderson, 1992) because individuals leaving the sampled area permanently appear to the model as dead (this can be remedied if dead marked individuals are recovered from areas outside the immediate sampled area, see Burnham, 1993). Animals leaving the immediate vicinity of the sampling grid temporarily and thus becoming unavailable for detection (e.g., for the duration of a primary occasion) can bias estimates of demographic parameters. Considerable attention has been dedicated to this issue of temporary emigration (Gilroy, Virzi, Boulton, & Lockwood, 2012; Hines, Kendall, Nichols, & Thompson, 2003; Kendall et al., 1997; Pradel, Hines, Lebreton, & Nichols, 1997). None of these approaches, however, account for the fact that the location of an individual relative to the sampling array affects how likely it is to become unavailable to sampling due to movements. Open SCR models hold the promise to directly address these issues by explicitly incorporating information on the spatial location and movement of individuals into the model (Royle et al., 2014).

One essential component to incorporating the movement of individuals in open SCR models is related to the “activity centers” that animals are assumed to have within a primary period and how those centers change between primary periods. In open SCR applications, the activity centers have been assumed to be constant over primary periods (Gardner et al., 2010; Whittington & Sawaya, 2015), changing randomly by primary period (Royle et al. 2014), or following a Gaussian random walk (Raabe et al., 2014; Schaub & Royle, 2014). The specification of the movement of activity centers among primary periods is one of the key mechanisms in distinguishing between emigration and survival. While in closed SCR models, the state space is set large enough such that density is invariant to its size, in open SCR applications, the state space also has to allow for the movement of activity centers between primary periods. Conceptually, however, the larger the state space, the more space individuals have to move and become unavailable to sampling. Thus, when individuals are not captured often in consecutive primary periods, then between‐primary‐period movements may be difficult to assess. In these cases, open SCR models may have confounding between movement and survival, and confounding may be particularly severe when activity centers are assumed to be independent between years.

We investigate the relationship of the movement model and state‐space delineation on survival and density estimates in open SCR models through a simulation study. To the best of our knowledge, simulations of open SCR models have been analyzed using the same state space and movement model as used in the data‐generating process (Gardner et al., 2010; Whittington & Sawaya, 2015) and performed well under these circumstances. However, in real‐life applications, the exact extent of the state space is rarely known. In addition to this simulation study, we apply open SCR models to a 10‐year camera‐trapping dataset of tigers (*Panthera tigris*) from Nagarahole Reserve in southern India (Karanth, 1995, Karanth, Nichols, Kumar, & Hines, 2006) to demonstrate potential issues in analysis of real field data when information on between‐year movement is sparse. Based on these results, we make recommendations for implementing open SCR models and for areas of future research and development.

## 2 MATERIALS AND METHODS

### 2.1 Model

To construct the model, let $y_{i,j,t}$ be the encounter history for individual *i*, at trap *j*, during primary occasion *t*. Due to the nature of how the data were recorded in the case study on tigers (maximum of one detection per camera per trap day for an individual tiger), we used a Binomial observation model in the data analysis and in the simulations. Thus, $\;y_{i,j,t}$ is defined such that

$$y_{i,j,t}|z_{i,t}∼\text{Binomial}\left(p\left(x,s\right)z_{i,t},K_{j,t}\right)$$

where, $K_{j,t}$ is the number of days trap *j* was active in primary occasion *t*, and $z_{i,t}$ is a binary indicator of whether individual *i* is alive at time *t* (note that we do not include any occasion, *k*, specific effects on detection in our model, and therefore, model counts $y_{\mathrm{ijt}}=\sum_{k}y_{i,j,k,t}$; but if there was any reason to suspect temporal effects on detection, this additional dimension could readily be introduced). We defined the encounter probability, $p\left(x,s\right),$ as

$$p\left(x,\;s\right)=1-\exp\left(\lambda_{t}\exp\left(\frac{-d{\left(x,s\right)}^{2}}{2\sigma_{p}^{2}}\right)\right)$$

where $\lambda_{t}$ is the baseline encounter probability at year *t* and $\sigma_{p}$ is the parameter that defines the rate of decline in detection as a function of distance, $d\left(x,s\right)$, from trap ***x*** to activity center ***s***.

Analogous to closed population SCR models implemented in a Bayesian framework, we used data augmentation (Royle et al., 2014) and estimated *z*_*i,t*_ for unobserved and augmented (hypothetical) individuals; *N*_*t*_ is then estimated as $\sum_{i}z_{i,t}$. For t≥2, $z_{i,t}∼\text{Bernoulli}\left(\phi z_{i,t-1}+\gamma_{t}\alpha_{i,t}\right)$; $\alpha_{i,t}$ is an indicator of whether an individual is available to be recruited or not. Thus, if $z_{i,t-1}=1$, then the individual can survive with probability ϕ and if $\alpha_{i,t}=1$, then an individual can be recruited with probability $\gamma_{t}$. This parameterization follows Gardner et al. (2010), where the recruitment parameters are interpreted as “conditional entrance probabilities,” not per‐capita recruitment. In this formulation of the open SCR model, the entrance probability is conditional on how many individuals in the augmented dataset are available to be recruited (i.e., have never been alive before); thus, γ should always be time/primary period specific. Per‐capita recruitment can be derived by dividing the number of recruits at time t, $R_{t},$ by $N_{t-1}$.

In closed population SCR models, activity centers can follow a homogeneous or inhomogeneous point process in the state‐space ***S***; in open population SCR, we have to consider if and how activity centers change between primary sampling periods. We explore three models describing movement of activity centers between primary periods: (a) Constant: Activity centers are held constant across years ($s_{t}=s$). This may be an appropriate model when dealing with highly site‐attached species that do not move much over their lifetimes. In this scenario, any shifting in activity centers will be “absorbed” into the parameter describing movement within primary periods (Royle, Fuller, & Sutherland, 2016). (b) Random/Independent: Activity centers are drawn from a uniform distribution across ***S*** each primary period ($s_{t}∼\text{Uniform}\left(S\right)$). This model assumes a complete spatial remixing of the population at each primary period (Royle et al., 2014). Whereas this is not an ecologically realistic representation of spatial population dynamics, the model is flexible by not forcing any specific relationship of movement between primary periods. Further, in exploratory analyses, we found this model to require less data to converge when compared to the Markovian movement model (see 3). As this model does not impose any constraint on movement, we expect that confounding of movement and survival may be severe; (c) Markovian: Activity center locations follow a Gaussian random walk (Raabe et al., 2014; Schaub & Royle, 2014) across primary periods. In this case, upon first entry into the population ($f_{i}$), individual activity centers are uniformly distributed across the state space, $s_{i,f_{i}}∼\text{Uniform}\left(S\right).$ At each subsequent primary period, $t\geq f_{i}$, the activity centers are modeled according to Gaussian random walk such that

$$s_{i,t}∼\text{Normal}\left(s_{i,\;t-1},\sigma_{s}^{2}I\right)$$

where ***I*** is the identity matrix. Here, $\sigma_{s}^{2}$ is the variance of the random walk (the variance increases as the number of primary periods increases), which is different from $\sigma_{p}^{2}$, the scale parameter in the encounter probability model defined above (related to movement within primary occasion). Note that the random walk is truncated at the limits of ***S***, which define the spatial domain of the model. This specification of the random walk seems like a logical choice when animals may be moving and shifting their activity center over time.

### 2.2 Simulation study

To investigate the sensitivity of the open population SCR model to changes in the size of the state space under different movement specifications, we performed a simulation study using the formulation of the open model described above (we describe an open SCR model for estimating only survival [SCR—Cormack–Jolly–Seber model] and provide full details of a comparable simulation study in Appendix S1). For all cases, we simulated *T *=* *5 years of population data, maintaining an average population size *N* of 40 individuals in a 10 × 10 unit state‐space *S*. In the center of *S* we placed a 7 × 7 sampling grid with a spacing of 1 unit. Animals survived with probability ϕ=0.75. To maintain average population size as constant, we created *M *=* *150 potential individuals and calculated annual conditional recruitment probability $\gamma_{t}$ as $N_{t-1}$ minus the number of survivors at *t*, divided by the number of potential individuals available for recruitment (i.e., individuals out of *M* that were never alive before *t*). The within‐year scale parameter $\sigma_{p}$ was 0.5; baseline detection $\lambda_{0}$ was 0.5, and each year had *K *=* *5 sampling occasions.

To explore the effects of changing the state space under different movement models, we created a 3 × 3 factorial design. First, we selected three movement models: constant activity centers over years, independent activity centers between years, and Markovian, using the random walk model described in the *Model* section, with variance parameter $\sigma_{s}^{2}=0.25$. We used these movement models for both data generation and analysis. Then, we selected three different buffer sizes (3*σ*_*p*_, 4*σ*_*p*_, and 5*σ*_*p*_) to define the state space in the analysis. We generated all datasets using a buffer of 4*σ*_*p*_, which means we analyzed the data using the same state space as was used to create them, plus one larger and one smaller state space. We implemented each of these nine scenarios (all combinations of buffer sizes and movement models) to explore potential differences between the model specifications.

We generated 100 datasets for each scenario described above, and we present the average posterior mean estimates, the relative root‐mean‐square error, bias, and 95% Bayesian confidence interval (BCI) coverage of the true value of all parameters across all 100 datasets. We used relatively noninformative priors for all parameters (see model specification in Appendix S5). We fitted all models in a Bayesian framework using JAGS (Plummer, 2003) through the rjags package (Plummer, 2016) in R 3.3.0 (R Core Team 2016). For each model, we ran three parallel chains with a 500 iteration burn‐in phase and 10000‐70000 posterior samples, thinned by 2.

### 2.3 Case Study: Tigers in Nagarahole reserve, India

Tiger camera‐trap data used in this study come from a study implemented in the central part of Nagarahole Reserve, in the state of Karnataka, southwestern India. The camera‐trap study was initiated in 1991, and we analyze data from 1991 to 2000, which had previously been analyzed using nonspatial capture–recapture models (Karanth et al., 2006). We note that there were 10 primary periods over 9 years; thus, sampling was not evenly spaced each year, and in the model, we estimate survival as $\phi^{\Delta t}$ and report annual survival as described in Karanth et al. (2006). The number of camera‐trap stations sampled per primary period ranged from 6 to 80. Sampling efforts initially covered only a 41.4 km^2^ area and were gradually expanded to 231.8 km^2^. Field method details are given in Karanth and Nichols (1998) and Karanth et al. (2006). Nonspatial capture–recapture estimates showed density varying considerably among years, from about 7 to over 20 individuals per 100 km^2^ (Karanth et al., 2006). Annual survival was 0.77 (*SE* 0.051), and there was evidence for temporary emigration and transiency in the population. Further details on the case study are provided in Appendix S4. We analyzed the data similarly to the simulation study, using a series of buffers (10, 15, and 18 km, corresponding to ~4, 6.5, and 8 times $\sigma_{p}$) and the three different movement models.

In the model implementation, we held $\sigma_{p}$, the scale parameter of the encounter probability model, constant across all 10 years of the study for two reasons: (a) We had no reason to assume that movement of individuals would be different between years, and (b) we fitted closed SCR models to each year and found very little variation in $\sigma_{p}$. For simplicity, we also held the baseline encounter rate $\lambda_{0}$ constant across years. The Karanth et al. (2006) model was complex and included transience and temporary emigration; for further comparison, we fitted a simpler nonspatial model with constant survival and constant detection over all primary periods.

We fitted all models in a Bayesian framework using JAGS (Plummer, 2003) through the rjags package (Plummer, 2016) in R 3.3.0 (R Core Team 2016). Results, reported as posterior summaries, are based on three chains each with 25,000–50,000 iterations and a burn‐in period of 2000. Convergence was assessed using the Gelman–Rubin statistic, $\hat{R}$ (Gelman & Rubin, 1992).

## 3 RESULTS

### 3.1 Simulation study

Detailed results for all open SCR model simulations are provided in Appendix S2. Results from the simulation study where the data were generated and analyzed with constant activity centers across all five primary periods indicated that survival was not biased when fitted under the three different state‐space sizes (Table 1). Under an independent activity centers model, estimates of survival showed low negative bias (7%) when the analysis state space was smaller than the data‐generating one, were essentially unbiased (<2%) when state spaces were identical, and had low positive bias (3%) when the analysis state space was larger than the data‐generating one. Coverage was below nominal (80%) only for the smaller state‐space scenario. When data were generated using a Markovian between‐year movement model, estimates of survival were not sensitive to specification of the state space (bias at or <1%, nominal coverage for all scenarios).

**Table 1:** Mean and relative root‐mean‐square error (rRSME) of the posterior means of survival (ϕ), based on 100 simulated datasets for each open population spatial capture–recapture model

Activity center model	3*σ* buffer		4*σ* buffer		5*σ* buffer	
	Mean	rRSME	Mean	rRSME	Mean	rRSME
Constant	0.75	0.06	0.75	0.06	0.75	0.07
Independent	0.70	0.10	0.74	0.06	0.77	0.07
Correlated	0.74	0.06	0.75	0.06	0.75	0.06

Notes

Three different buffer sizes (columns) were used to delineate the state space (4*σ* is data‐generating state space) and combined with three different models for how activity centers change over primary period (rows). Data‐generating value of ϕ=0.75

Under the constant activity centers model, density estimates ranged from 0.41 to 0.43 for all years and all state‐space sizes (Table A4). The bias was low, but showed a slight pattern of increasing as the state‐space size increased, from 1%–3% in the smallest to 6%–9% in the largest state space (Table A4). Density estimates for the independent activity centers model exhibited low to moderate positive bias (3%–12%) and below nominal coverage (75%–91%) when the analysis state space was smaller than the generating state space. Density estimates were essentially unbiased (1%–4%) and had (sometimes just below) nominal coverage when the state space was the same for data generation and analysis. Lastly, density estimates showed low negative bias (1%–3%) and nominal coverage when the analysis state space was larger than the data‐generating state space (Table A5).

Under a Markovian between‐year movement model, density estimates ranged from 0.40 to 0.43 for all years and all state‐space sizes (Table A6). The bias in density estimates was similar to the constant activity center model with slightly lower bias in smallest state space (2%–4%) and increasing to 6‐8% in the largest state space. Overall for the Markovian model, the confidence interval coverage was nominal, except when the analysis state space was smaller than the data‐generating state space (coverage 80%–87%; Table A6).

Detection parameters ($\lambda_{0},\;\sigma_{p}$) were essentially unbiased (relative bias <1.5%) under the all three movement models and had nominal 95% BCI coverage, with the exception of $\lambda_{0},$ which only had about 90% coverage under the Markovian movement model (Table A6) and slightly higher (91%–94%) coverage under the constant model (Table A4). In the Markovian movement model, the parameter $\sigma_{s}$ had an average posterior mean estimate of 0.49 across all state‐space sizes and therefore had a relative bias that was slightly negative (−2%) [Table A6]. The recruitment parameters were not directly compared as they are relative to the study area size and data augmentation parameter. All results are based on the posterior mean, which was similar to the mode as the posterior distributions were generally not skewed, but we note that the mode may be less biased and should be considered when posterior distributions are skewed.

### 3.2 Case Study

Of the 75 individuals in the tiger camera‐trap dataset, 42 individuals were only observed in one year of the study; one individual was known to be present for the entire duration of the study (recaptured in six primary periods), one for 8 years, and all others between 2 and 6 years. Of those individuals observed in multiple years, 14 had gaps in their annual encounter histories, that is, were observed at *t* and t+x, where *x *>* *1, but not at t+1. In cases with the correlated activity center model, sparse data resulted in difficulty reaching convergence particularly for $\sigma_{s}^{2}$, but this appeared to have little impact on estimates of the other parameters.

**Figure 1:**
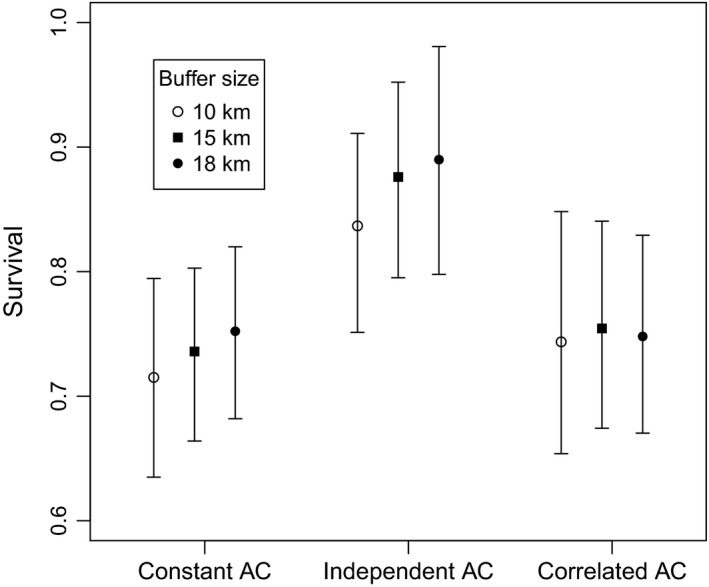
Posterior mean estimates of tiger survival from Nagarahole reserve, India, based on camera‐trapping data from 1991 to 2000 using open spatial capture–recapture models with three different activity center models (Constant AC, Independent AC, and Correlated AC), and three different buffer sizes used to delineate the state space (10 km, 15 km, and 18 km); bars represent 95% Bayesian credible intervals

**Figure 2:**
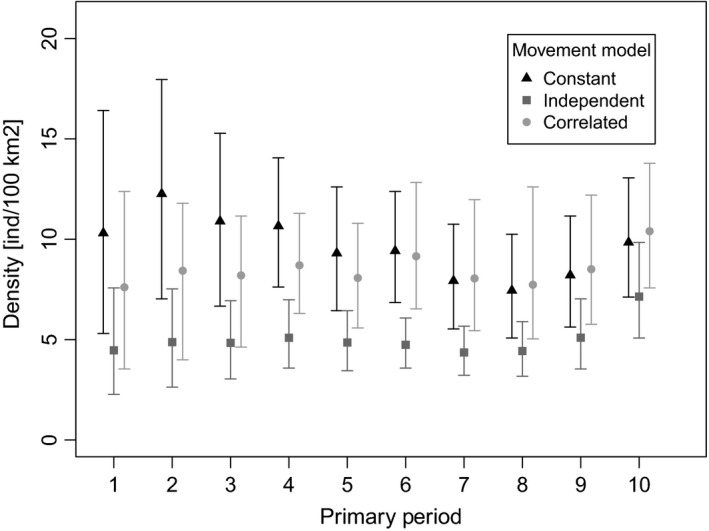
Estimates of tiger density (individuals/100 km^2^) from Nagarahole reserve, India, based on camera‐trapping data from 1991 to 2000 using open spatial capture–recapture models; bars represent 95% Bayesian credible intervals. Three different models for the activity centers are shown: constant activity centers in triangles, independent activity centers in squares, and correlated activity centers in circles. Results are shown for a state space based on a 15‐km trap array buffer

Analysis of the dataset with an open SCR model with three different buffer sizes (10, 15, and 18 km) and three different activity center models (constant, independent, and Markovian) resulted in posterior mean estimates of survival ranging from 0.72 to 0.89 (Figure 1). The lowest estimates of survival were under the constant activity center model with the smallest buffer size (0.72 ± 0.04). Increasing the buffer size in the constant activity center model increased the estimated survival (0.75 ± 0.04), though not substantially. The same pattern was seen with the independent activity center model, where survival was lowest in the 10 km buffer (0.84 ± 0.04) and increased as the buffer size increased (15 km buffer: 0.88 ± 0.04; 18 km buffer: 0.89 ± 0.05). Survival estimates were relatively constant under the correlated activity center model (10 km buffer: 0.74 ± 0.05; 15 km buffer: 0.75 ± 0.04; 18 km buffer: 0.75 ± 0.04). Density estimates for each of the 10 years varied over the different model specifications as well, ranging from 4.1 to 13.9 tigers/100 km^2^ with the estimates of density lowest for all primary periods for the independent AC model and highest in the first six primary periods for the constant AC model (Figure 2).

## 4 DISCUSSION

By making efficient use of available data and explicitly incorporating animal movement in the estimation of survival, open SCR models hold promise as a framework for monitoring and assessment of wildlife population dynamics. Their biggest appeal is perhaps the modeling of temporary or permanent emigration, in the form of explicit models for how animal home range (or use area) location changes over time. As advances in survey techniques allow more studies to collect capture–recapture data across larger time periods, the need for a framework to analyze such data is also growing. The results of our simulation study demonstrate, however, that estimates of survival and density from open SCR models are sensitive to the definition of the state space and the model describing movement of individual activity centers between years.

In the simulation study, the constant activity center model generally performed well. The low positive bias in density (between 1 and 9%) increased slightly with increasing state‐space sizes (Table A4 in Appendix S2). Survival estimates under this model were constant and only slightly (1%) negatively biased (that bias increased slightly to ‐3% for the CJS version of the model; Table A1); however, in the case study, we found that the posterior mean survival estimates increased as the state‐space size was increased when holding activity centers constant across primary periods. The contrasting results are likely due to misspecification of the activity center model in the tiger case study, with the data indicating that tiger activity centers did change over time (discussed below).

The model in which animal activity centers were independent of each other across years was the most sensitive to specification of the state space. This was reflected in both the simulation study and the tiger case study. Not surprisingly, we observed the same pattern of bias in survival in the CJS formulation of the model (Appendix S1, Table A2). As ***S*** increases, it provides increasing amounts of area for animals to remain alive but unavailable to sampling. Contrary to the Markovian movement model and the constant activity center model, the independent model contains no component constraining animal movement between years; the model is therefore unable to parse out movement off the sampling grid from mortality and produces increasingly positively biased estimates of survival as ***S*** increases (and negatively biased estimates as ***S*** decreases). Bias in density estimates was mostly minor under this model, but showed the opposite pattern (positive bias in smaller ***S*** and vice versa), in both the simulation study (Table A5 in Appendix S2) and the tiger case study (Figure 2).

The Markovian model performed much better in simulations (including for the CJS version of the model, Table A3) than the independent model, even though there seemed to be consistent (albeit low) positive bias in estimates of density, which appeared to increase with increasing ***S*** (Table A6 in Appendix S2).

We only considered simple movement models in our simulation study, and real populations are almost inevitably more complex in their behavior. For example, tiger populations typically consist of residents and “floaters” (individuals that do not hold a territory, Smith, 1993); such groups can differ in their spatial behavior across years. Similarly, none of the models considered here adequately accounts for transient individuals, even though their presence could bias estimates of demographic parameters, particularly in the constant and correlated activity center models, which restrict between‐year movement of individuals. Further, we only explored scenarios with uniform density across ***S***. In heterogeneous landscapes, more complex movement models taking into account variation in permeability (Sutherland et al., 2015) or habitat suitability (Royle et al., 2013) may be warranted. Considering the sensitivity of parameter estimates to specification of the movement model in the simpler models considered here, performance of more complex models and their data requirements need to be explored with great care.

### 4.1 Tiger case study

The case study dataset of tigers in Nagarahole presented here was previously analyzed with a nonspatial open population model by Karanth et al. (2006). Consistent with the simulation study, under the independent activity center model estimated survival increased as the state space increased (Figure 1). The estimated survival under the independent activity center model was much higher (0.84–0.89) than under the constant activity center (0.72–0.75) and correlated activity center models (0.74–0.75), as well as the nonspatial model ($\hat{\phi }=0.77$) of Karanth et al. (2006) and a simple nonspatial model with constant detection and survival ($\hat{\phi }=0.71$). When we implemented a constant activity center model, the survival rate increased as the state space increased (see Figure 1), a different pattern from the simulation results where survival did not change with the state‐space size. This is likely because the constant activity center model does not adequately represent the underlying process of spatial population dynamics (we do not expect all tigers to have constant activity centers over 10 years). A number of tigers were captured only once over the course of the study; therefore, if the state space increases, those animals’ activity centers can be assumed to be located further from the trap array, which decreases their detection probability, and that, in turn, allows them to have a higher survival probability. A summary of the parameter estimates for all nine scenarios is provided in Table A9.

Even though the Markovian activity center model probably does not fully reflect the true underlying spatial dynamics of the tiger population, which is characterized by residents, transients, and temporary emigrants (Karanth et al., 2006), estimates of survival probability were fairly constant under that model when size of ***S*** changed (Figure 1). Estimates of density showed sensitivity to the size of ***S*** in early years of the study, but were consistent in later years, when more data were available. The independent activity center model also consistently led to lower density estimates compared with the two other models. For both survival and density, these between‐model differences in estimates were more pronounced in the tiger data analysis than in the simulation study. Unfortunately, in the tiger dataset, convergence of the variance parameter of the Markov process was extremely slow to unattainable. This is likely due to a lack of consistent recaptures of individuals across subsequent years, and consequently, little available information on how much activity centers shift from year to year. The tiger dataset may be a somewhat extreme case study, as the area covered by traps varied in size considerably across years and was very small in some of the early years, thus reducing the number of individuals exposed to potential recapture. It is conceivable that incorporation of telemetry data (Sollmann et al., 2013) may improve the ability to model between‐year movements by providing detailed information on individual location, even if off the sampling grid.

Karanth et al. (2006) presented nonspatial model densities, which ranged from 7.33 to 21.73 individuals/100 km^2^, compared to 4.1 to 6.83 individuals/100 km^2^ in the independent activity center model with an 18 km buffer and 7.39 to 13.9 individuals/100 km^2^ in the constant activity center model with a 10 km buffer. For lack of knowledge of true density, we compared open model densities to estimate from closed population SCR models fitted to seven primary periods of the study (Appendix S4, Table A10). The independent activity center model estimates of tiger density were all lower than those from closed SCR models, whereas the other two models yielded density estimates similar to those from closed SCR models. The overall higher nonspatial density estimates are likely a methodological artifact. Several studies have shown that SCR models yield lower density estimates for wide‐ranging animals because of their improved ability to account for movement off the sampling grid (O'Connell et al., 2011; Tobler & Powell, 2013). This discrepancy is exacerbated in early years of the study, when the small area covered with camera‐traps did not capture tiger movements adequately, leading to underestimation of the effective sampled area and consequently, overestimation of density (Karanth et al., 2006).

Annual spatial density estimates of the Nagarahole tiger population were mostly lower but on the same order of magnitude as estimates based on single‐year data from a survey implemented across the entire reserve (including areas surveyed in earlier years) in 2006 of ~13 individuals/100 km^2^ (Royle et al., 2009). Even though this study does not coincide temporally with and spans a larger area than the data analyzed here, we note it because Royle et al. (2009) used a habitat mask to exclude nontiger habitat from the state space, which reduced the area in which tigers can occur by about 50%. One of our objectives was to discuss the impact of changing the state‐space size in open SCR models, and using a habitat mask is another way to effectively resize the state space. It is possible that restrictive habitat masks may cause inflated density estimates; careful specification of habitat masks is important in SCR modeling, and in some cases, it may be better to include information on habitat suitability in the model for detection probability rather than exclude habitat categorically (Royle et al., 2014). Inclusion of a habitat mask in an open SCR model is conceptually straightforward; however, careful thought about the mask and potential changes in habitat must consider prior to analysis and sensitivity of parameter estimates to the habitat mask should be conducted.

### 4.2 Recommendations for using open SCR models

The present study exposed some potential challenges with the implementation of open SCR models, but broader exploration of model behavior under different situations is warranted. Based on this study, we conclude that if the state space is known (e.g., the case of an island, or suitable habitat in a clearly defined matrix), open SCR models perform well, across various options for modeling animal movement between years. We performed a reduced simulation study to investigate the effects of misspecifying the between‐year movement model, Appendix S3, but recommend further testing of model misspecification.

When the data‐generating state space is known and sampled exhaustively, the independent activity center model is a flexible movement model that produces largely unbiased estimates of survival and density, even when the true underlying movement model is more complex (Markovian, or Markovian with occasional longer distance movements, Appendix S3). In all other situations, however, we do not recommend using a model with independent activity centers over multiple year studies, due to its sensitivity to the definition of ***S***.

The constant activity center model performed well in simulations, which is similar to the findings of Royle et al. (2016) who found that in terms of estimating density, the constant model was unbiased in closed SCR models. However, the tiger data analysis suggested that ignoring movement of activity centers over time may result in sensitivity of parameter estimates to specification of ***S***. For most species, it seems unrealistic to assume no change in activity centers over longer time frames. A comprehensive simulation study of the constant model should be conducted to determine the robustness of this model to longer time series and different movement patterns between primary periods.

The Markovian random walk model also performed well in the simulation study. However, we found in the tiger data analysis that the model achieved convergence very slowly, likely because of limited between primary period movement information. Thus, if a dataset contains insufficient information to estimate the parameters of the Markov process, we suggest using other available information (telemetry data, published information on dispersal), or the detection data on recaptured individuals, to construct an informative prior, or, if necessary, fix the Markov process variance parameter. We did not explore the performance of either one of these approaches in the present study, and in such cases, we recommend exploring sensitivity of the survival and density estimates to the specification of the prior/parameter.

## 5 CONCLUSION

Explicitly incorporating space into capture–recapture models is generally stated as an advantage of SCR models, bearing the promise of more realistic representation of animal populations, and improved estimates of parameters describing these populations. Open SCR models can easily accommodate study designs that vary within and between primary periods, as shown in our case study analysis of tiger population dynamics. These advantages to SCR modeling are promising as the need for estimating demographic rates remains an essential component in ecological studies. For open SCR models, specification of the movement of individual activity centers between primary periods is an important component of model fitting. In all cases, we suggest careful thought be given to the movement of activity centers between primary periods and the limitations outlined in this study.

## AUTHORS’ CONTRIBUTION

BG and RS designed the simulation study, analyzed the data, and led the writing. NSK and KUK designed the tiger study and collected the data. All authors contributed to the concept, critically reviewed drafts, and gave final approval for publication.

## DATA ACCESSIBILITY

Data available on the Dryad Digital Repository.
