# Supplementary material for: State space and movement specification in open population spatial capture–recapture models
Source: Ecol Evol. 2018 Sep 27;8(20):10336–44. doi: 10.1002/ece3.4509 (PMC6206188; doi:10.1002/ece3.4509)
Supplement: Supplementary file 1 [file ECE3-8-10336-s001.pdf]

SCR-CJS data simulation and model fitting example

SCR-CJS Simulation with Correlated Activity Centers

Here we show how to simulate data (sim.fn) under a correlated activity center model, create the jags model file for the same model ("CJS.mod"), and then fits the data to the model.

First, we create jags specification to fit the model.

```
cjs.mod <- "model { #model

# Priors

phi ~ dunif(0,1)        # Survival (constant)
sigP ~ dunif(0,10)      # Intercept in sigma estimate
sigP2<-sigP*sigP
lam0 ~ dgamma(0.1, 0.1)  # Encounter rate
sigS~dunif(0,10)
tauXY <- 1/(sigS * sigS)

for (i in 1:M){ #m

       z[i,first[i]] <- 1  # Known to be alive at entry into study
      SX[i,first[i]] ~ dunif(xl, xu)
      SY[i,first[i]] ~ dunif(yl, yu)

      for(j in 1:J){
           D2[i,j,first[i]] <- pow(SX[i,first[i]]-trapmat[j,1], 2) + pow(SY[i,first[i]]-trapma
t[j,2],2)
            g[i,j,first[i]] <- lam0*exp(-D2[i,j,first[i]]/(2*sigP2))
        pmean[i,j,first[i]] <- 1- exp(-g[i,j,first[i]])
          tmp[i,j,first[i]] <- z[i,first[i]]*pmean[i,j,first[i]]
            y[i,j,first[i]] ~ dbin(tmp[i,j,first[i]], K)
        }

   for (t in (first[i]+1):T) { #t
             SX[i,t]~dnorm(SX[i, t-1], tauXY)T(xl,xu)  # set priors for the X and Y coordinate
s of each individual
             SY[i,t]~dnorm(SY[i, t-1], tauXY)T(yl,yu)
          phiUP[i,t]<- z[i,t-1]*phi
                z[i,t] ~ dbern(phiUP[i,t])

       for(j in 1:J){
           D2[i,j,t] <- pow(SX[i,t]-trapmat[j,1], 2) + pow(SY[i,t]-trapmat[j,2],2)
            g[i,j,t] <- lam0*exp(-D2[i,j,t]/(2*sigP2))
        pmean[i,j,t] <- 1- exp(-g[i,j,t])
          tmp[i,j,t] <- z[i,t]*pmean[i,j,t]
            y[i,j,t] ~ dbin(tmp[i,j,t], K)
        }
```

```
      } # t
  } # m
} #model"
```

There are built in R functions to calculate the distance between multiple points, but we used this older function:

```
e2dist <- function (x, y)
{   i <- sort(rep(1:nrow(y), nrow(x)))
    dvec <- sqrt((x[, 1] - y[i, 1])^2 + (x[, 2] - y[i, 2])^2)
    matrix(dvec, nrow = nrow(x), ncol = nrow(y), byrow = F)
}
```

Next, write a function to simulate the data.

```
sim.fn <-
function(N,phi0,lam0,M, T, grid, xl, xu, yl, yu, sigP, K, sigS){ # M is total ever alive

ntraps<- dim(grid)[1]
nreps<- K
lam0<-rep(lam0,T)
phi<-rep(phi0,T)
pmat<-lam<-list()

gamma<-NULL
gamma[1]<- N/M

sx<-sy<-sin<-matrix(NA, nrow=M, ncol=T)
sx[,1]<-runif(M,xl,xu)
sy[,1]<-runif(M,yl,yu)

z<-r<-matrix(0,nrow=M,ncol=T)
r[,1]<-rbinom(M,1,gamma[1])
z[,1]<-r[,1]


for (t in 2:T){

sx[,t]<-runif(M,xl,xu)
sy[,t]<-runif(M,yl,yu)

surv<-rbinom(M,1,z[,t-1]*phi[t])

#corr acitvity centers for guys alive before
al<-apply(matrix(z[,1:(t-1)],nrow=M,byrow=FALSE),1,sum)>0
sx[al,t]<-rtnorm(sum(al),sx[al,t-1],sigS, lower=xl, upper=xu)
sy[al,t]<-rtnorm(sum(al),sy[al,t-1],sigS, lower=yl, upper=yu)


idx<- 1- as.numeric(al)
gamma[t]<- (N - sum(surv))/sum(idx)
if (gamma[t]<0) gamma[t]<-0
```

```
 r[,t]<- rbinom(M,idx,gamma[t])
 z[,t]<- surv + r[,t]
}

for (t in 1:T){
S<-cbind(sx[,t], sy[,t])

dmat<-e2dist(S,grid)
psi<- exp(-(1/(2*sigP*sigP))*dmat*dmat)
lam[[t]]<- lam0[t]*psi
pmat[[t]]<- 1- exp(-lam[[t]])
}

y<-array(0,dim=c(M,ntraps,T))

for(t in 1:T){
   yfull<-array(0,dim=c(M,ntraps,K))

for(i in 1:M){
for (k in 1:K) {
yfull[i,1:ntraps,k]<-rbinom(ntraps,1, pmat[[t]][i,]*z[i,t] )
}
}
y[, 1:ntraps,t]<-apply(yfull,1:2,sum)
}

ycapt=y[which(rowSums(y[,,])>0),,]
sx=sx
sy=sy

#calcuate the period of first capture
first=NULL
capsums<-apply(ycapt, 3, rowSums)
for(i in 1:dim(ycapt)[1]){
a=which(capsums[i,] >0)
first[i] = min(a)
}

list(y=ycapt,r=r,gamma=gamma,N=apply(z,2,sum),R=apply(r,2,sum), first=first, SX=sx, SY=sy, al=
al, N=dim(ycapt)[1])
}
```

Be sure to install the rjags package if you have not already. Also, to use the rtnorm command, we need the msm package.

```
library(msm)
```

```
## Warning: package 'msm' was built under R version 3.5.1
```

```
library(rjags)
```

```
## Warning: package 'rjags' was built under R version 3.5.1
```

```
## Loading required package: coda
```

```
## Linked to JAGS 4.2.0
```

```
## Loaded modules: basemod,bugs
```

Now all of the parameters and the state space can be set for the simulation and analysis.

```
#set up the basic trap array in a 7x7 grid
gridx<-seq(-3,3,1)
grid<-as.matrix(expand.grid(gridx,gridx))
J=dim(grid)[1]
#set the upper and lower x and y coordinates for the state space
xl<- -5
yl<- -5
xu<-5
yu<-5

T<-K<-5 #number of years/seasons

sigP=0.5
lam0=0.5
N=40
M=150
phi0=0.75
sigS=0.5

#function to creat initial S for each year
Sin<-function(first=first,T=T, M=M, xl=xl, xu=xu, yl=yl, yu=yu,ntot=ntot){
SX<-SY<-matrix(NA, nrow=ntot, ncol=T)

for(i in 1:ntot){
 for (t in simdat$first[i]:T){
  SX[i,t]<-runif(1, xl, xu)
  SY[i,t]<-runif(1, yl, yu)

 traps<-which(simdat$y[i,,t]>0)
 if(length(traps)>0){
 SX[i,t]<- mean(grid[traps,1])
 SY[i,t]<- mean(grid[traps,2])
}
}
}

return(list(SX, SY))
}
```

Use the simulation function from above

```
set.seed(1028)
```

```
simdat<-sim.fn(N=N,lam0=lam0, phi0=phi0,M=150, T=T, grid=grid, xl=xl, xu=xu, yl=yl, yu=yu, sig
P=sigP, K=K,sigS=sigS)
```

Set up the variables and intial values needed for jags

```
#set up intial values for z
zin<-matrix(1, dim(simdat$y)[1], T)
for(i in 1:dim(simdat$y)[1]){
for(t in 1:simdat$first[i]){
zin[i,t]<-NA
}}

#statespace is 4x, as is data generation
data<-list(y=simdat$y, first=simdat$first, M=dim(simdat$y)[1],T=5,xl=xl, xu=xu, yl=yl, yu=yu,K
=K, J=J, trapmat=as.matrix(grid))

inits =  function() {list(phi=runif(1, .5, 1),sigP=runif(1,1,2), lam0=runif(1), z=zin, sigS=ru
nif(1,0.1,3),
        SX=Sin(first=simdat$first,T=T, xl=xl, xu=xu, yl=yl, yu=yu, ntot=dim(simdat$y)[1])[[1]]
,
        SY=Sin(first=simdat$first,T=T, xl=xl, xu=xu, yl=yl, yu=yu, ntot=dim(simdat$y)[1])[[2]]
) }

params<-c("phi","sigP", "sigS", "lam0")
```

Run the model. Here we supply very small values for the adaptation and number of iterations just for an example (as computation time can be quite cumbersome). You should select larger values or the ones in the manuscript for running this model.

```
cjs.mod2 <- textConnection(cjs.mod)
mod<-jags.model(cjs.mod2, data, inits, n.chains=3, n.adapt=100)
out<-coda.samples(mod, params, n.iter=100, thin=3)
close(cjs.mod2)
```
