# Supplementary material for: State space and movement specification in open population spatial capture–recapture models
Source: Ecol Evol. 2018 Sep 27;8(20):10336–44. doi: 10.1002/ece3.4509 (PMC6206188; doi:10.1002/ece3.4509)
Supplement: Supplementary file 2 [file ECE3-8-10336-s002.pdf]

SCJ - JS data simulation and model fitting example

SCR-JS Simulation with Constant Activity Centers

Here we show how to simulate data (sim.fn) under a constant activity center model, create the jags model file for the same model (“js.mod”), and then fit the data to the model.

First, we create jags specification to fit the model.

```
js.mod <- "model { #model

# set priors for sigma2, lam0 (encounter rate), gamma, and phi
sigma~dunif(0, 10)
sigma2 <- sigma*sigma
lam0 ~ dunif(0,5)
phi~dunif(0, 1)     #survival

for(t in 1:T){             # T = 10 years
    gamma[t]~dunif(0, 1)   #recruitment
    N[t]<-sum(z[1:M,t])    #N per year
   Rc[t]<-sum(R[1:M,t])    #number recruited

     for (i in 1:M){            #loop over M individuals (includes the augmented data)
    for(j in 1:J) {    #loop over all traps of that year
            D2[t,i,j] <- pow(SX[i]-trapmat[j,1], 2) + pow(SY[i]-trapmat[j,2],2)
           g[t,i,j] <- lam0*exp(-D2[t,i,j]/(2*sigma2))
        pmean[t,i,j] <- 1- exp(-g[t,i,j])
          tmp[t,i,j] <- z[i,t]*pmean[t,i,j]
           y[t,i,j] ~ dbin(tmp[t,i,j],K)  #K is the number of days a trap was operational
        }
     }
   }

for (i in 1:M){
      z[i,1]~dbin(gamma[1], 1)
      a[i,1]<-(1-z[i,1])
      R[i,1]<- z[i,1]      #Calculate recruits
      SX[i]~dunif(xl, xu)  # set priors for the X and Y coordinates of each individual
      SY[i]~dunif(yl, yu)

  for(t in 2:T){

      a1[i,t] <- sum(z[i, 1:t])          #have you ever been alive (0 = no, >1 = yes)
       a[i,t] <- 1-step(a1[i,t] - 1)     #use the step function to make a1 binary
      mu[i,t] <- (phi*z[i,t-1]) + (gamma[t]*a[i,t-1])
       z[i,t]~dbern(mu[i,t])
       R[i,t]<-z[i,t]*a[i,t-1]
          }
}
```

```
} #model"
```

There are built in R functions to calculate the distance between multiple points, but we used this older function:

```
e2dist <- function (x, y)
{   i <- sort(rep(1:nrow(y), nrow(x)))
    dvec <- sqrt((x[, 1] - y[i, 1])^2 + (x[, 2] - y[i, 2])^2)
    matrix(dvec, nrow = nrow(x), ncol = nrow(y), byrow = F)
}
```

Next, write a function to simulate the data.

```
simJS.fn <-
function(N,phi0,lam0,M, T, grid, xl, xu, yl, yu, sigma, K){ # M is total ever alive

ntraps<- dim(grid)[1]
nreps<- K
lam0<-rep(lam0,T)
phi<-rep(phi0,T)

pmat<-lam<-list()
gamma<-NULL
gamma[1]<- N/M

sx<-runif(M,xl,xu)
sy<-runif(M,yl,yu)

z<-r<-al<-matrix(0,nrow=M,ncol=T)
r[,1]<-rbinom(M,1,gamma[1])
z[,1]<-r[,1]


for (t in 2:T){
surv<-rbinom(M,1,z[,t-1]*phi[t])

#recruitment
al[,t]<-apply(matrix(z[,1:(t-1)],nrow=M,byrow=FALSE),1,sum)>0
idx<- 1- as.numeric(al[,t])
gamma[t]<- (N - sum(surv))/sum(idx)
if(gamma[t]<0) gamma[t]<-0

 r[,t]<- rbinom(M,idx,gamma[t])
 z[,t]<- surv + r[,t]
}

S<-cbind(sx, sy)
dmat<-e2dist(S,grid)
psi<- exp(-(1/(2*sigma*sigma))*dmat*dmat)
for (t in 1:T){
lam[[t]]<- lam0[t]*psi
pmat[[t]]<- 1- exp(-lam[[t]])

}
```

```
y<-array(0,dim=c(M,ntraps,T))
for(t in 1:T){
yfull<-array(0,dim=c(M,ntraps,K))

for(i in 1:M){
for (k in 1:K) {
yfull[i,1:ntraps,k]<-rbinom(ntraps,1, pmat[[t]][i,]*z[i,t] )
}
}
y[, 1:ntraps,t]<-apply(yfull,1:2,sum)
}

ycapt=y[which(rowSums(y[,,])>0),,]

list(y=ycapt, z=z,r=r,gamma=gamma,N=apply(z,2,sum),R=apply(r,2,sum), SX=sx, SY=sy)
}
```

Be sure to install the rjags package if you have not already.

```
library(rjags)
```

```
## Warning: package 'rjags' was built under R version 3.5.1
```

```
## Loading required package: coda
```

```
## Linked to JAGS 4.2.0
```

```
## Loaded modules: basemod,bugs
```

Now all of the parameters and the state space can be set for the simulation and analysis.

```
#set up the basic trap array in a 7x7 grid
gridx<-seq(-3,3,1)
grid<-as.matrix(expand.grid(gridx,gridx))
J=dim(grid)[1]

#set the upper and lower x and y coordinates for the state space
xl<- -5
yl<- -5
xu<-5
yu<-5
T<-K<-5 #number of years/seasons

sigma=0.5
lam0=0.5
N=40
M=150
phi0=0.75
tau=0.5
```

```
#function to creat initial S for each individual for jags
Sin<-function(T=T, M=M, xl=xl, xu=xu, yl=yl, yu=yu,ntot=ntot){
SX<-SY<-matrix(NA, nrow=M, ncol=1)

for(i in 1:M){
for (t in 1:T){
SX[i]<-runif(1, xl, xu)
SY[i]<-runif(1, yl, yu)

traps<-which(dataug[i,,t]>0)
if(length(traps)>0){
SX[i]<- mean(grid[traps,1])
SY[i]<- mean(grid[traps,2])
}
}
}

return(list(SX, SY))
}
```

Use the simulation function from above

```
Mc<-150 #upper M for data augmentation

#simulate data based on simJS.fn
simdat<-simJS.fn(N=N,phi0=phi0,lam0=lam0,M=Mc, T=T, grid=grid, xl=xl, xu=xu, yl=yl, yu=yu, sig
ma=sigma, K=K)
```

Set up the variables and intial values needed for jags

```
ntot=dim(simdat$y)[1] #total ever observed in this simulated dataset

#add Mc-ntot zero encounter histories (data augmentation)
dataug<-array(0, dim=c(Mc, J, T))
dataug[1:ntot,,]<-simdat$y
dataugTMJ<-aperm(dataug, c(3,1,2))

#create intial values for z state
zinit<-matrix(0,nrow=Mc, ncol=T)
zinit[1:ntot,]<-1


data<-list(M=Mc,y=dataugTMJ, T=T, J=J, xl=xl, xu=xu, yl=yl, yu=yu, trapmat=as.matrix(grid), K=
K)

inits =  function() {list(phi=runif(1), gamma=runif(T,0,1),sigma=runif(1, 1,2),z=zinit,
        lam0=runif(1), SY=as.vector(Sin(T=T, M=Mc, xl=xl, xu=xu, yl=yl, yu=yu, ntot=ntot)[[2]]
),
        SX=as.vector(Sin(T=T, M=Mc, xl=xl, xu=xu, yl=yl, yu=yu,  ntot=ntot)[[1]])) }

params<-c("phi","sigma","lam0","N", "Rc","gamma")
```

Run the model. Here we supply very small values for the adaptation and number of iterations just for an example (as computation time can be quite cumbersome). You should select larger values or ones in the manuscript for running this model.

```
js.mod2 <- textConnection(js.mod)
mod<-jags.model(js.mod2, data, inits, n.chains=3, n.adapt=100)
out<-coda.samples(mod, params, n.iter=100, thin=1)
close(js.mod2)
```
