# Supplementary material for: State space and movement specification in open population spatial capture–recapture models
Source: Ecol Evol. 2018 Sep 27;8(20):10336–44. doi: 10.1002/ece3.4509 (PMC6206188; doi:10.1002/ece3.4509)
Supplement: Supplementary file 3 [file ECE3-8-10336-s003.docx]

**Appendix 1:**

*Spatial Capture-Recapture Cormack-Jolly-Seber Simulation study:*

Model Description. We conducted a simulation study for exploring the sensitivity of parameters to changes in the size of the state space under different movement specifications in a Cormack-Jolly-Seber version of an SCR model. The model was constructed similarly to the JS model described in the main text, where $y_{i,j,t}$ is the encounter history for individual $i$, at trap $j$, during primary occasion $t$. We used a Binomial observation model such that

$$y_{i,j,t}|z_{i,t}\sim\mathrm{Binomial}(p\left( x,s \right), K_{j,t})$$

where $, K_{j,t}$ is the number of days trap *j* was active in primary occasion $t$, and $z_{i,t}$is a binary indicator of whether individual $i$ is alive at time $t$. We conditioned on an animal’s first capture period, $f(i)$*,* and set the alive state,$z_{i,j,f(i)}=1$. For$t>f(i)$, $z_{i,t}\sim Bernoulli(\phi z_{i,t-1})$; $t$hus if $z_{i,t-1}=1$, then the individual can survive with probability $\phi$.

As with the JS model implementation, we defined the encounter probability as

$$p\left( \boldsymbol{x, s} \right)=1-{\exp\left( \lambda_{t}\exp\left( \frac{-d\left( \boldsymbol{x,s} \right)^{2}}{2\sigma_{p}^{2}} \right) \right)}$$

where $\lambda_{t}$ is the baseline encounter probability at year $t$ and $\sigma_{p}$ is the parameter that defines the rate of decline in detection as a function of distance, $d\left( \boldsymbol{x}\boldsymbol{,}\boldsymbol{s} \right)$, from trap $\boldsymbol{x}$ to activity center $\boldsymbol{s}.$We simulated data under three different models for the movement of activity centers between years. First, a constant model where an individual’s activity center is constant over the entire study, $\boldsymbol{s}_{i}\sim\mathrm{Uniform}\left( \boldsymbol{S} \right)$. Second, an independent model where an individual’s activity center for each primary period can vary but is not directly related to other primary periods, $\boldsymbol{s}_{i,t}\sim\mathrm{Uniform}\left( \boldsymbol{S} \right)$. Third, a correlated model such that an individual’s activity center is uniformly distributed across the state space upon first entry into the population, $\boldsymbol{s}_{i,f_{i}}\sim\mathrm{Uniform}\left( \boldsymbol{S} \right)$ and at each subsequent primary period, $t\geq f_{i}$, the activity centers are modeled according to Gaussian random walk such that

$$\boldsymbol{s}_{i,t}\sim\mathrm{Normal}(\boldsymbol{s}_{i, t-1},\sigma_{s}^{2}\boldsymbol{I})$$

where $I$ is the identity matrix. Here, $\sigma_{s}^{2}$ is the variance of steps in the the random walk (quantifying the amount activity centers shift between primary occasions), which is different from $\sigma_{p}^{2}$, the scale parameter in the encounter probability model defined above (quantifying within primary occasion movement).

It is important to note that while the Cormack-Jolly-Seber models condition on first capture and thus does not estimate detection probability for the first primary period, we specify this spatial CJS model differently. Specifically, we allow detection to be estimated in the first primary period for two main reasons: 1) The data from SCR type surveys provide spatial capture information from that first primary period that can be used to estimate detection (as in the Robust Design) and 2) for the Gaussian random walk model, we want to estimate the activity center in the first primary period so that the activity center for the second primary period can be estimated based on the first period.

We simulated data exactly as described in the simulation study for the Jolly-Seber model. For all cases, we simulated *T*=5 years of population data, maintaining an average population size *N* of 40 individuals in a 10x10 unit state space $S$. In the center of $S$ we placed a 7x7 sampling grid with a spacing of 1 unit. Animals survived with probability $\phi= 0.75$. To maintain average population size as constant, we created *M* = 150 potential individuals and calculated annual conditional recruitment probability $\gamma_{t}$ as $N_{t-1}$ minus the number of survivors at $t$, divided by the number of potential individuals available for recruitment (i.e., individuals out of *M* that were never alive before $t$). The within-year movement parameter $\sigma_{p}$ was 0.5; baseline detection $\lambda_{0}$ was 0.5, and each year had *K* = 5 sampling occasions.

We used the same 3 by 3 factorial design for the simulation study with the 3 different movement models described above and 3 different buffer sizes ($3\sigma, 4\sigma, \mathrm{and} 5\sigma$) to define the state space in the analysis. We generated all datasets using a buffer of $4\sigma$, which means we analyzed the data using the same buffer as was used to create it and then one larger and one smaller. In an analysis of real data, the “true” state space is generally unknown. We implemented each of these 9 scenarios (all combinations of buffer size and movement model) to explore potential differences between the demographic model specifications.

We generated 100 datasets for each scenario described above. For each of the scenarios, we present the average estimates, the relative root mean square error, bias, and 95% Bayesian Confidence Interval coverage of the true value of all parameters across all 100 datasets. For each model, we ran three parallel chains with a 250 iteration burn-in phase and 10000 posterior samples, thinned by 3.

*Results*

The constant activity center model had similar estimates across the different buffer sizes; however, survival was underestimated in all three scenarios. Survival estimates declined with increasing $S$ under the independent activity center movement model, and were mostly unbiased under the data generating $S$. A similar but weaker pattern was observable under the correlated activity center model.

Table A1: Parameter estimates from CJS-SCR model with constant activity centers for *T*=5 years ($\phi$=survival, $\sigma$=scale parameter of the detection function, $\lambda_{0}$= baseline detection) over 100 simulated datasets for three different state space sizes (data generating is 4$\sigma$**)**. This table shows the average posterior mean, relative root mean square error (rRMSE), relative bias (rB), and 95% Bayesian Confidence Interval coverage (Cov). Data generating values: $\lambda_{0}=0.5, \sigma=0.5, \phi=0.75.$

| $\boldsymbol{S}$ | **Parameter** | **mean** | **RMSE** | **rB** | **Cov** |
| --- | --- | --- | --- | --- | --- |
| **5** $\boldsymbol{\sigma}$ | $\lambda_{0}$ | 0.49 | 0.09 | -0.01 | 0.95 |
|  | $\phi$ | 0.73 | 0.07 | -0.02 | 0.96 |
|  | $\sigma$ | 0.51 | 0.04 | 0.03 | 0.87 |
|  |  |  |  |  |  |
| **4**$\boldsymbol{\sigma}$ | $\lambda_{0}$ | 0.51 | 0.09 | 0.02 | 0.95 |
|  | $\phi$ | 0.73 | 0.08 | -0.03 | 0.89 |
|  | $\sigma$ | 0.51 | 0.04 | 0.02 | 0.92 |
|  |  |  |  |  |  |
| **3**$\boldsymbol{\sigma}$ | $\lambda_{0}$ | 0.51 | 0.09 | 0.02 | 0.95 |
|  | $\phi$ | 0.73 | 0.08 | -0.03 | 0.89 |
|  | $\sigma$ | 0.51 | 0.04 | 0.02 | 0.93 |

Table A2: Parameter estimates from the CJS- SCR model with independent activity centers for *T*=5 years ($\phi$=survival, $\sigma$=scale parameter of the detection function, $\lambda_{0}$= baseline detection) over 100 simulated datasets for three different state space sizes (data generating is 4$\sigma$**)**. This table shows the average posterior mean, relative root mean square error (rRMSE), relative bias (rB), and 95% Bayesian Confidence Interval coverage (Cov). Data generating values: $\lambda_{0}=0.5, \sigma=0.5, \phi=0.75.$

| $\boldsymbol{S}$ | **Parameter** | **mean** | **RMSE** | **rB** | **Cov** |
| --- | --- | --- | --- | --- | --- |
| **5** $\boldsymbol{\sigma}$ | $\lambda_{0}$ | 0.50 | 0.08 | 0.01 | 0.98 |
|  | $\phi$ | 0.79 | 0.08 | 0.05 | 0.85 |
|  | $\sigma$ | 0.52 | 0.04 | 0.03 | 0.89 |
|  |  |  |  |  |  |
| **4** $\boldsymbol{\sigma}$ | $\lambda_{0}$ | 0.50 | 0.08 | 0.01 | 0.98 |
|  | $\phi$ | 0.74 | 0.07 | -0.01 | 0.94 |
|  | $\sigma$ | 0.51 | 0.04 | 0.03 | 0.91 |
|  |  |  |  |  |  |
| **3** $\boldsymbol{\sigma}$ | $\lambda_{0}$ | 0.50 | 0.08 | 0.01 | 0.98 |
|  | $\phi$ | 0.70 | 0.10 | -0.07 | 0.83 |
|  | $\sigma$ | 0.51 | 0.04 | 0.02 | 0.95 |

Table A3: Parameter estimates from the CJS- SCR model with correlated activity centers for *T*=5 years ($\phi$=survival, $\sigma$=scale parameter of the detection function, $\lambda_{0}$= baseline detection) over 100 simulated datasets for three different state space sizes (data generating is 4$\sigma$**)**. This table shows the average posterior mean, relative root mean square error (rRMSE), relative bias (rB), and 95% Bayesian Confidence Interval coverage (Cov). Data generating values: $\lambda_{0}=0.5, \sigma=0.5, \phi=0.75.$

| $\boldsymbol{S}$ | **Parameter** | **mean** | **RMSE** | **rB** | **Cov** |
| --- | --- | --- | --- | --- | --- |
| **5**$\boldsymbol{\sigma}$ | $\lambda_{0}$ | 0.50 | 0.08 | 0.00 | 0.97 |
|  | $\phi$ | 0.74 | 0.07 | -0.02 | 0.95 |
|  | $\sigma$ | 0.51 | 0.05 | 0.03 | 0.90 |
|  |  |  |  |  |  |
| **4**$\boldsymbol{\sigma}$ | $\lambda_{0}$ | 0.50 | 0.08 | 0.00 | 0.97 |
|  | $\phi$ | 0.74 | 0.07 | -0.02 | 0.94 |
|  | $\sigma$ | 0.51 | 0.04 | 0.03 | 0.91 |
|  |  |  |  |  |  |
| **3**$\boldsymbol{\sigma}$ | $\lambda_{0}$ | 0.50 | 0.08 | 0.01 | 0.97 |
|  | $\phi$ | 0.72 | 0.08 | -0.03 | 0.93 |
|  | $\sigma$ | 0.51 | 0.04 | 0.03 | 0.91 |

**Appendix 2**

*Detailed results of simulation study for Jolly-Seber formulation of the open model described in detail in the main text*

We simulated *T*=5 years of population data, maintaining an average population size *N* of 40 individuals in a 10x10 unit state space $S$. In the center of $S$ we placed a 7x7 sampling grid with a spacing of 1 unit. Animals survived with probability $\phi= 0.75$. The within-year movement parameter $\sigma_{p}$ was 0.5; baseline detection $\lambda_{0}$ was 0.5, and each year had *K* = 5 sampling occasions.

We simulated data using three movement models: constant activity centers over years, independent activity centers between years, and correlated activity centers between years using the random walk model, with variance parameter $\sigma_{s}^{2}=0.25$. We analyzed each dataset under the generating movement model using 3 different buffer sizes ($3\sigma_{p}, 4\sigma_{p}, \mathrm{and} 5\sigma_{p}$) to define the state space. We generated 100 datasets for each of the 9 scenarios, and tables below present the average estimates, the relative root mean square error, bias, and 95% Bayesian Confidence Interval coverage of the true value of all parameters across all 100 datasets (Table A5: constant activity centers; Table A6: independent activity centers, Table A7: Markovian movement of activity centers).

Table A4: Parameter estimates from open SCR model with constant activity centers ($\boldsymbol{s}_{t}\sim\mathrm{Uniform}(S)$) for *T*=5 years (*D* = density [individuals/unit^2^], $\phi$=survival, $\sigma_{p}$=within year movement parameter, $\lambda_{0}$= baseline detection) over 100 simulated datasets, using 3 different buffers to define the state space S for analysis (data generating S used $4\sigma_{p}$buffer). Showing the posterior mean, relative root mean square error (rRMSE), relative bias (rB), and 95% Bayesian Confidence Interval coverage (Cov).

| $\boldsymbol{S}$ | **Parameter** | **Mean** | **rRMSE** | **rB** | **Cov** |
| --- | --- | --- | --- | --- | --- |
| **5**$\boldsymbol{\sigma}$ | *D_1_^*^* | 0.42 | 0.12 | 0.06 | 0.99 |
|  | *D_2_^*^* | 0.43 | 0.13 | 0.08 | 0.98 |
|  | *D_3_^*^* | 0.43 | 0.12 | 0.08 | 0.99 |
|  | *D_4_^*^* | 0.43 | 0.13 | 0.09 | 0.96 |
|  | *D_5_^*^* | 0.43 | 0.13 | 0.07 | 0.97 |
|  | $\lambda_{0}$ | 0.51 | 0.09 | 0.02 | 0.95 |
|  | $\phi$ | 0.75 | 0.07 | -0.01 | 0.93 |
|  | $\sigma_{p}$ | 0.50 | 0.03 | 0.00 | 0.98 |
|  |  |  |  |  |  |
| **4**$\boldsymbol{\sigma}$ | *D_1_^*^* | 0.41 | 0.11 | 0.03 | 0.99 |
|  | *D_2_^*^* | 0.42 | 0.12 | 0.05 | 0.98 |
|  | *D_3_^*^* | 0.42 | 0.10 | 0.05 | 0.99 |
|  | *D_4_^*^* | 0.42 | 0.12 | 0.06 | 0.97 |
|  | *D_5_^*^* | 0.42 | 0.13 | 0.05 | 0.95 |
|  | $\lambda_{0}$ | 0.51 | 0.09 | 0.02 | 0.95 |
|  | $\phi$ | 0.75 | 0.06 | -0.01 | 0.93 |
|  | $\sigma_{p}$ | 0.50 | 0.03 | -0.01 | 0.98 |
|  |  |  |  |  |  |
| **3**$\boldsymbol{\sigma}$ | *D_1_^*^* | 0.40 | 0.11 | 0.01 | 0.94 |
|  | *D_2_^*^* | 0.41 | 0.11 | 0.03 | 0.90 |
|  | *D_3_^*^* | 0.41 | 0.10 | 0.03 | 0.93 |
|  | *D_4_^*^* | 0.41 | 0.12 | 0.03 | 0.89 |
|  | *D_5_^*^* | 0.41 | 0.12 | 0.03 | 0.90 |
|  | $\lambda_{0}$ | 0.51 | 0.10 | 0.02 | 0.94 |
|  | $\phi$ | 0.75 | 0.06 | -0.01 | 0.93 |
|  | $\sigma_{p}$ | 0.50 | 0.04 | 0.00 | 0.98 |

*Average data generating density was 0.4 individuals/unit^2^; rRMSE and rB and Cov calculated using the replicate-specific value of *D*.

Table A5: Parameter estimates from open SCR model with independent activity centers ($\boldsymbol{s}_{t}\sim\mathrm{Uniform}(S)$) for *T*=5 years (*D* = density [individuals/unit^2^], $\phi$=survival, $\sigma_{p}$=within year movement parameter, $\lambda_{0}$= baseline detection) over 100 simulated datasets, using 3 different buffers to define the state space S for analysis (data generating S used $4\sigma_{p}$buffer). Showing the posterior mean, relative root mean square error (rRMSE), relative bias (rB), and 95% Bayesian Confidence Interval coverage (Cov).

| $\boldsymbol{S}$ | **Parameter** | **Mean** | **rRMSE** | **rB** | **Cov** |
| --- | --- | --- | --- | --- | --- |
| **5**$\boldsymbol{\sigma}$ | *D_1_^*^* | 0.39 | 0.12 | -0.01 | 0.95 |
|  | *D_2_^*^* | 0.39 | 0.09 | -0.03 | 0.96 |
|  | *D_3_^*^* | 0.39 | 0.08 | -0.02 | 0.95 |
|  | *D_4_^*^* | 0.38 | 0.10 | -0.03 | 0.98 |
|  | *D_5_^*^* | 0.39 | 0.11 | -0.02 | 0.97 |
|  | $\lambda_{0}$ | 0.50 | 0.09 | -0.00 | 0.97 |
|  | $\phi$ | 0.77 | 0.06 | 0.03 | 0.94 |
|  | $\sigma_{p}$ | 0.50 | 0.03 | 0.00 | 0.96 |
|  |  |  |  |  |  |
| **4**$\boldsymbol{\sigma}$ | *D_1_^*^* | 0.39 | 0.12 | 0.01 | 0.94 |
|  | *D_2_^*^* | 0.41 | 0.10 | 0.02 | 0.94 |
|  | *D_3_^*^* | 0.41 | 0.09 | 0.04 | 0.96 |
|  | *D_4_^*^* | 0.40 | 0.09 | 0.01 | 0.95 |
|  | *D_5_^*^* | 0.40 | 0.11 | 0.01 | 0.96 |
|  | $\lambda_{0}$ | 0.50 | 0.09 | -0.01 | 0.97 |
|  | $\phi$ | 0.74 | 0.06 | -0.02 | 0.99 |
|  | $\sigma_{p}$ | 0.50 | 0.03 | -0.00 | 0.97 |
|  |  |  |  |  |  |
| **3**$\boldsymbol{\sigma}$ | *D_1_^*^* | 0.40 | 0.13 | 0.03 | 0.91 |
|  | *D_2_^*^* | 0.44 | 0.14 | 0.10 | 0.75 |
|  | *D_3_^*^* | 0.44 | 0.13 | 0.12 | 0.64 |
|  | *D_4_^*^* | 0.43 | 0.12 | 0.08 | 0.79 |
|  | *D_5_^*^* | 0.41 | 0.12 | 0.03 | 0.88 |
|  | $\lambda_{0}$ | 0.49 | 0.09 | -0.01 | 0.96 |
|  | $\phi$ | 0.70 | 0.10 | -0.07 | 0.80 |
|  | $\sigma_{p}$ | 0.50 | 0.04 | -0.01 | 0.96 |

*Average data generating density was 0.4 individuals/unit^2^; rRMSE and rB and Cov calculated using the replicate-specific value of *D*.

Table A6: Parameter estimates from open SCR model with correlated activity centers ($\boldsymbol{s}_{t}\sim\mathrm{Normal}(s_{t-1},\sigma_{s}^{2}\boldsymbol{I})$) for *T*=5 years (*D* = density [individuals/unit2], $\phi$=survival, $\sigma_{s}$= $standard deviation of the step length in Markovian random walk, \sigma_{p}= scale parameter in the detection function, \lambda_{0}$= baseline detection) over 100 simulated datasets, with a $4\sigma_{p}$buffer to define the data generating state space S. Showing mean, relative root mean square error (rRMSE), relative bias (rB), and 95% Bayesian Confidence Interval coverage (Cov).

| $\boldsymbol{S}$ | **Parameter** | **Mean** | **rRMSE** | **rB** | **Cov** |
| --- | --- | --- | --- | --- | --- |
| **5**$\boldsymbol{\sigma}$ | *D_1_^*^* | 0.43 | 0.14 | 0.07 | 0.98 |
|  | *D_2_^*^* | 0.43 | 0.13 | 0.06 | 0.98 |
|  | *D_3_^*^* | 0.43 | 0.13 | 0.08 | 0.96 |
|  | *D_4_^*^* | 0.43 | 0.13 | 0.07 | 0.95 |
|  | *D_5_^*^* | 0.42 | 0.13 | 0.06 | 0.99 |
|  | $\lambda_{0}$ | 0.50 | 0.11 | 0.00 | 0.89 |
|  | $\phi$ | 0.75 | 0.06 | 0.01 | 0.96 |
|  | $\sigma_{s}$ | 0.49 | 0.12 | -0.02 | 0.94 |
|  | $\sigma_{p}$ | 0.50 | 0.04 | -0.00 | 0.96 |
|  |  |  |  |  |  |
| **4**$\boldsymbol{\sigma}$ | *D_1_^*^* | 0.42 | 0.13 | 0.05 | 0.98 |
|  | *D_2_^*^* | 0.42 | 0.12 | 0.04 | 0.98 |
|  | *D_3_^*^* | 0.42 | 0.12 | 0.05 | 0.94 |
|  | *D_4_^*^* | 0.42 | 0.12 | 0.04 | 0.96 |
|  | *D_5_^*^* | 0.41 | 0.12 | 0.03 | 0.99 |
|  | $\lambda_{0}$ | 0.50 | 0.11 | 0.01 | 0.89 |
|  | $\phi$ | 0.75 | 0.06 | 0.00 | 0.96 |
|  | $\sigma_{s}$ | 0.49 | 0.12 | -0.02 | 0.95 |
|  | $\sigma_{p}$ | 0.50 | 0.04 | -0.00 | 0.96 |
|  |  |  |  |  |  |
| **3**$\boldsymbol{\sigma}$ | *D_1_^*^* | 0.41 | 0.12 | 0.04 | 0.87 |
|  | *D_2_^*^* | 0.41 | 0.12 | 0.02 | 0.83 |
|  | *D_3_^*^* | 0.41 | 0.11 | 0.03 | 0.84 |
|  | *D_4_^*^* | 0.41 | 0.12 | 0.03 | 0.8 |
|  | *D_5_^*^* | 0.40 | 0.12 | 0.02 | 0.86 |
|  | $\lambda_{0}$ | 0.50 | 0.11 | 0.00 | 0.89 |
|  | $\phi$ | 0.74 | 0.06 | -0.01 | 0.97 |
|  | $\sigma_{s}$ | 0.49 | 0.12 | -0.02 | 0.93 |
|  | $\sigma_{p}$ | 0.50 | 0.04 | -0.00 | 0.96 |

*Average data generating density was 0.4 individuals/unit^2^; rRMSE and rB and Cov calculated using the replicate-specific value of *D*.

**Appendix 3**

*Simulation study: mis-specification of the movement model*

To investigate the sensitivity of the open population SCR model to mis-specification of the model for movement between primary periods, we performed a small additional simulation study. We simulated *T*=5 years of population data, maintaining an average population size *N* of 30 individuals in a 9x9 unit state space $S$. In the center of $S$ we placed a 7x7 sampling grid with a spacing of 1 unit. Animals survived with probability $\phi= 0.75$. To maintain average population size constant, we created *M* = 150 potential individuals and calculated annual conditional recruitment probability $\gamma_{t}$ as $N_{t-1}$ minus the number of survivors at $t$, divided by the number of potential individuals available for recruitment (i.e., individuals out of *M* that were never alive before $t$). The within-year movement parameter $\sigma_{p}$ was 0.5; baseline detection $\lambda_{0}$ was 0.5, and each year had *K* = 5 sampling occasions.

When first recruited into the population, individual activity centers $s_{i{,f}_{i}}$ were drawn from a uniform distribution over $S$; in Scenario 1, in subsequent years, $s$ was drawn according to a Gaussian random walk with mean $s_{i,t-1}$ and variance $\sigma_{s}^{2} = {0.5}^{2}$, truncated to the extent of the state space. In Scenario 2, each year an animal had a probability of 0.25 to potentially make a long-distance movement between $t- 1$ and *t*; in that case, $s_{it}$ was drawn from Gaussian random walk with mean $s_{i,t-1}$and variance $\sigma_{s, disp}^{2} = {2.5}^{2}$.

We generated 100 datasets for each scenario and analyzed them with an open population SCR model in which activity centers were assumed to be randomly distributed across $S$ every year (i.e., they are independent across years). For each of the two scenarios, we present the average estimates, the relative root mean square error, bias, and 95% Bayesian Confidence Interval coverage of the true value of all parameters across all 100 datasets. We implemented this additional simulation study as described for the main simulation study; for each model we ran three parallel chains with a 500 iteration burn-in phase and 10000 posterior samples, thinned by 2.

Results

Analysis of the dataset with an open SCR model with temporally correlated activity centers but analyzed with a mis-specified model with random (i.e., uncorrelated) activity centers, showed largely unbiased estimates of $\phi$ (<1% positive bias), as well as of $\sigma$ and $\lambda_{0}$ (1 and 2% bias, respectively), with nominal ($\phi$) or close to nominal ($\sigma$, $\lambda_{0}$) credible interval coverage (Table A7). Estimates of $N$ were unbiased in year 1 and 5 (<1% negative bias), but showed some negative bias in intermediate years, ranging from 3 to 6% (Table A7). Coverage was below or close to nominal for estimates of $N$ (between 0.83 and 0.88, Table A7).

In Scenario 2, when simulated activity centers were correlated and animals had a 0.25 chance of making a long distance movement within *S*, the random activity center model returned unbiased estimates of $\sigma$ (<1%, see Table A8). The estimate of $\lambda_{0}$ showed low positive bias (2%), and the estimate of $\phi$ showed low negative bias (2%, Table A8); all three parameters had nominal credible interval coverage. Except for year 2 and 3 (<1% negative bias), estimates of $N$ showed low positive bias (2 to 3%, Table A8). Coverage was below or close to nominal for estimates of $N$ (0.78 to 0.91, Table A8).

Table A7: Results from the simulation study for Scenario 1. Data were simulated using the random walk activity centers and fitted with the independent activity center model. We present the true parameter estimate, the mean, the root mean square error (RMSE), bias, and coverage for abundance *N*, survival $\phi$, baseline encounter rate $\lambda_{0}$ and movement parameter $\sigma$.

| **Parameter** | **True** | **Mean** | **RMSE** | **Bias** | **Coverage** |
| --- | --- | --- | --- | --- | --- |
| *N_1_* | 30.02 | 29.38 | 0.16 | -0.01 | 0.85 |
| *N_2_* | 29.99 | 28.47 | 0.14 | -0.05 | 0.84 |
| *N_3_* | 30.34 | 28.49 | 0.14 | -0.06 | 0.83 |
| *N_4_* | 30.27 | 29.27 | 0.13 | -0.03 | 0.85 |
| *N_5_* | 30.21 | 30.25 | 0.12 | 0.00 | 0.88 |
| $\lambda_{0}$ | 0.5 | 0.51 | 0.11 | 0.02 | 0.94 |
| $\phi$ | 0.75 | 0.76 | 0.06 | 0.01 | 0.95 |
| $\sigma$ | 0.5 | 0.51 | 0.04 | 0.01 | 0.91 |

Table A8: Results from the simulation study for Scenario 2. Data were simulated using the random walk activity centers additionally, each year an animal had a probability of 0.25 to potentially make a long distance movement, and fitted with the independent activity center model. We present the true parameter estimate, the mean, the root mean square error (RMSE), relative bias, and coverage for abundance *N*, survival $\phi$, baseline encounter rate $\lambda_{0}$ and movement parameter $\sigma$.

| **Parameter** | **True** | **Mean** | **RMSE** | **Bias** | **Coverage** |
| --- | --- | --- | --- | --- | --- |
| *N_1_* | 30.06 | 30.54 | 0.18 | 0.03 | 0.78 |
| *N_2_* | 30.28 | 30.23 | 0.12 | 0.00 | 0.85 |
| *N_3_* | 30.07 | 29.97 | 0.11 | 0.00 | 0.91 |
| *N_4_* | 29.75 | 30.09 | 0.13 | 0.02 | 0.84 |
| *N_5_* | 29.97 | 30.54 | 0.18 | 0.03 | 0.78 |
| $\lambda_{0}$ | 0.5 | 0.51 | 0.09 | 0.02 | 0.97 |
| $\phi$ | 0.75 | 0.74 | 0.07 | -0.02 | 0.94 |
| $\sigma$ | 0.5 | 0.50 | 0.03 | 0.00 | 0.99 |

**Appendix 4**

*Case Study of Tigers in Nagarahole reserve, India*

The unique stripe patterns of tigers allow for individual identification from photographs. As a result, camera-traps can be used to generate individual encounter histories without the need to physically capture and tag animals (Karanth 1995, Karanth and Nichols 1998). The data used in this paper are from a study implemented in the central part of Nagarahole reserve, in the state of Karnataka, southwest India. The 644-km^2^ reserve is characterized by tropical deciduous forests and protects high-density prey and tiger populations (Karanth and Nichols 1998. Karanth et al. 2006). This camera-trap study originally started in 1991, we focus on data from 1991 to 2000, which were previously analyzed using non-spatial capture-recapture models (Karanth et al. 2006). Sampling effort varied over years and the number of camera trap stations (each with 2 cameras facing one another) ranged from 6-80 and the estimated sampled area ranged from 41.4 km^2^ area to 231.8 km^2^ (estimated sampled area is calculated as described in Karanth and Nichols (1998)). At the same time, sampling duration was reduced from 162 days in 1991 to 30-40 days in the later years of the study. Details concerning the field methods are given in Karanth and Nichols (1998) and Table 1 of Karanth et al. (2006) shows the primary periods, number of days sampled, the estimated sampled area, the effort in trap-nights, and the number of detected individuals. In the original analysis of the data set, records of 74 individuals collected over 5725 trap nights were included. Because data collected in later years allowed for the identification of additional individuals, the data set for the present analysis contained 75 individuals.

Results from the 9 fitted models are shown in Table A9. In the Markovian model, we note that the initial years of density did not converge and the parameter $\sigma_{s}$ was very slow to mix. While $\sigma_{s}$ mixed poorly, we did find consistent results in estimate this parameter across subsets of the data, for example when using only the last 5 primary periods, all parameters converged and the results are very similar to those reported here. We do note that N was not converged for the first two primary periods in the Markovian model and we recommend caution in considering these as true estimates of the density.

Non-spatial capture recapture estimates showed that the Nagarahole tiger population, on average, was increasing by about 3% annually, with density varying considerably among years, from about 7 to over 20 individuals per 100 km^2^ (Karanth et al. 2006). Annual survival was 0.77 (SE 0.051), and there was evidence for temporary emigration and transiency in the population. These previous analyses were unable to estimate trends in abundance over the entire course of the study due to variation in the sampled area; SCR models overcome this issue by explicitly modeling the spatial location of traps and movement of individuals. Thus we aim to provide consistent estimates of population size, as well as survival and recruitment, even during early years of the study when the data were sparse.

Karanth et al. (2006) found some indication of a behavioral response to camera-trapping and a considerable portion of transients in the population. We explored if there were any behavioral responses in analyzing single-year data sets with closed SCR models, but found little to no effect and thus we did not include a behavioral response in the open model analysis. We also assumed that the potential effect of transients would be addressed by allowing individuals to have activity centers that can move off the trap array; therefore, we did not include a separate survival parameter for transients as was done in Karanth et al. (2006).

Additionally, for further context and comparison of the open population SCR model estimates of tiger density, we fit closed population SCR models to single year camera-trapping data of tigers. We only fit models for years 1994 to 2000 (primary periods 4 -10), because of low sample size in earlier years of the study. We defined the state space as a given year’s trap array buffered by 15 km. We fit models using JAGS through R, as described for the main analyses in the main text; we ran three parallel MCMC chains with 5000 iterations burn-in and based inference on 10000 posterior samples. We confirmed convergence of parallel chains using the r-hat statistics (all r-hats < 1.02). Results are listed in Table A10.

Table A9 – Posterior mean results for each parameter from the full tiger analysis. Each model specification and state space buffer is shown (e.g., Const10 – Constant activity center model with a buffer size of 10km; Ind15 – Independent activity center model with a 15km buffer; Mark18 – Markovian activity center model with a buffer of 18km).

|  | Const10 | Const15 | Const18 | Ind10 | Ind15 | Ind18 | Mark10 | Mark15 | Mark18 |
| --- | --- | --- | --- | --- | --- | --- | --- | --- | --- |
| Density - 1991 | 11.39 | 10.31 | 8.34 | 5.29 | 4.46 | 4.10 | 9.21 | 7.61 | 7.50 |
| Density - 1991 | 13.90 | 12.26 | 9.95 | 5.74 | 4.87 | 4.48 | 10.69 | 8.43 | 8.24 |
| Density - 1992 | 11.93 | 10.90 | 9.25 | 5.52 | 4.84 | 4.56 | 10.06 | 8.20 | 8.08 |
| Density - 1992 | 11.31 | 10.66 | 9.24 | 5.78 | 5.09 | 4.78 | 10.14 | 8.70 | 9.14 |
| Density - 1995 | 9.61 | 9.31 | 8.32 | 5.34 | 4.86 | 4.62 | 8.98 | 8.07 | 8.33 |
| Density - 1996 | 9.58 | 9.42 | 8.71 | 5.20 | 4.74 | 4.51 | 9.42 | 9.15 | 9.20 |
| Density - 1997 | 7.96 | 7.93 | 7.52 | 4.54 | 4.36 | 4.20 | 8.00 | 8.05 | 7.88 |
| Density - 1998 | 7.39 | 7.45 | 7.10 | 4.54 | 4.43 | 4.30 | 7.43 | 7.73 | 7.44 |
| Density - 1999 | 8.43 | 8.21 | 7.78 | 5.50 | 5.09 | 4.93 | 8.43 | 8.51 | 8.17 |
| Density - 2000 | 10.68 | 9.84 | 9.08 | 7.76 | 7.14 | 6.83 | 10.66 | 10.40 | 9.92 |
| $\lambda_{0}$ | 0.02 | 0.02 | 0.02 | 0.03 | 0.03 | 0.03 | 0.03 | 0.03 | 0.03 |
| $\phi$ | 0.72 | 0.74 | 0.75 | 0.84 | 0.88 | 0.89 | 0.74 | 0.75 | 0.75 |
| $\sigma_{p}$ | 2.19 | 2.22 | 2.25 | 2.22 | 2.26 | 2.28 | 2.02 | 2.04 | 2.04 |
| $\sigma_{s}$ | -- | -- | -- | -- | -- | -- | 1.12 | 1.09 | 1.12 |

Table A10: Density estimates (posterior mean and standard deviation, individuals/100km^2^) from closed population spatial capture-recapture models fit to single-year camera trapping data of tigers from Nagarahole reserve, India, between 1994 and 2000. Parameters $\lambda_{0}$ and $\sigma$ were held constant over all primary periods and posterior mean and standard deviation for both are shown below.

| **Parameter** | **Mean** | **SD** |
| --- | --- | --- |
| Density - 1994 | 12.57 | 2.54 |
| Density - 1995 | 8.97 | 2.26 |
| Density - 1996 | 8.74 | 1.70 |
| Density - 1997 | 6.73 | 1.78 |
| Density - 1998 | 6.80 | 1.53 |
| Density - 1999 | 8.32 | 1.71 |
| Density - 2000 | 11.31 | 1.89 |
| $\lambda_{0}$ | 0.03 | 0.00 |
| $\sigma$ | 2.01 | 0.10 |

**Appendix 5**

1. Code to simulate data under the correlated activity centers model for the SCR-CJS model and code to fit the model. CJSRmarkdownExp.html file attached.
2. Code to simulate data under the constanct activity centers model for the SCR-JS model and code to fit the model. JSRmarkdownExp.htlm file attached.
